# Improving Mechanical Properties of PLA/Starch Blends Using Masterbatch Containing Vegetable Oil Based Active Ingredients

**DOI:** 10.3390/polym13172981

**Published:** 2021-09-02

**Authors:** Bianka Nagy, Norbert Miskolczi, Zoltán Eller

**Affiliations:** Research Centre of Biochemical, Environmental and Chemical Engineering, MOL Department of Hydrocarbon & Coal Processing, Faculty of Engineering, University of Pannonia, H-8200 Veszprém, Hungary; nagy.bianka@mk.uni-pannon.hu (B.N.); ellerz@almos.uni-pannon.hu (Z.E.)

**Keywords:** PLA/starch, compatibilizer, vegetable oil-based additive, masterbatch

## Abstract

The aim of this research was to increase the compatibility between PLA and starch with vegetable oil-based additives. Based on tensile results, it can be stated, that Charpy impact strength could be improved for 70/30 and 60/40 blends in both unconditioned and conditioned cases, regardless of vegetable oil, while no advantageous change in impact strength was obtained with PLA-g-MA. Considering sample with the highest starch concentration (50%), the flexural modulus was improved by using sunflower oil-based additive, Charpy impact strength and elongation at break was increased using rapeseed oil-based additive in both conditioned and unconditioned cases. SEM images confirmed the improvement of compatibility between components.

## 1. Introduction

The main challenges of the growing demand for petroleum-derived plastics are their long degradation periods, health risks, price volatility, waste disposal problems, and increasing demand for raw materials [1,2]. As a consequence, the development of biodegradable polymers from renewable sources has become increasingly conspicuous in recent years [2,3]. Biodegradable polymers can be converted to carbon dioxide, water, methane, and other products [4]. Many biodegradable polymers are known nowadays, such as polylactic acid (PLA), polycaprolactone (PCL), polybutylene adipate terephthalate (PBAT), polyhydroxybutyrate (PHB), and poly(hydroxyalkanoates) (PHA). Starch, known as a natural raw material, is also considered a promising alternative to biopolymers or their constituents [4].

Starch-containing polymers can be divided into four types: thermoplastic starch (TPS), starch/synthetic aliphatic polyester, starch/PBS or PBSA polyester, starch/PVOH [2]. Starch/biodegradable polymer blends are considered an auspicious method to improve the mechanical and thermal properties of native starch. Furthermore, due to their hydrophilic nature, the quality of starch-based blends also depend largely on their moisture content as well [4,5]. Blending the economically viable starch with PLA offers an attractive alternative. PLA generally has good mechanical properties, its strength and stiffness being comparable to, among others, polyethylene terephthalate (PET) and polystyrene (PS). However, its disadvantages include its fragility, its relatively slow rate of degradation in soil, and its higher production costs compared to petroleum-based polymers. By blending of PLA and starch, the good mechanical properties of PLA and the good biodegradability and low manufacturing cost of starch can be combined [6,7,8].

Most PLA-based plastic blends and composites show partial or complete incompatibility [9]. Hydrophobic PLA and hydrophilic starch are not thermodynamically miscible with each other resulting phase separation and weak interfacial adhesion in their blends. The lack of compatibility gives disadvantageous mechanical properties [7]. Thus, ensuring the compatibility of starch and PLA is essential to improve the mechanical properties [4,5]. During compatibilization, the agents are located at the interface reducing the interfacial tension and preventing the coalescence of the dispersed phase, improving the interfacial adhesion, and creating a thermodynamically stable structure [10]. There are basically four general methods for compatibilization: using copolymers, reactive compatibilization, using nanoparticles, and “radical” processing [10,11,12]. PLA-g-MA is a potential compatibilizer for PLA-based blends. In PLA/starch systems, interfacial adhesion can be improved by reducing the size of dispersed phase [13]. Another option is to use of bio-based agents. Vegetable oils provide a remarkable alternative instead of petroleum-derived additives with the result that their application is becoming more widespread [9,14]. Some fatty acids allow different chemical modifications due to their single or multiple unsaturation [14]. Modified vegetable oils can behave as a compatibilising agent in binary and ternary blends [9]. Now, epoxidized, MA-modified, and acrylated-epoxidized vegetable oils are known for industrial application [14].

The main motivation of this research is to focus on the production and application of additives based on vegetable oil (sunflower oil, rapeseed oil and castor oil) suitable for improving the miscibility properties of PLA/starch blends. The primary purpose was to produce a masterbatch with compatibilizing nature improving the mechanical properties of PLA/starch blends with different composition.

## 2. Materials and Methods

### 2.1. Materials

In this work corn starch (supplied by HungranaBioeconomy Company (Szabadegyháza, Hungary) was blended into commercial grade PLA (IngeoTM Biopolymer 4043D, Minnetonka, MN, USA) as matrix material. To achieve better interfacial properties of the PLA/starch blends, experimentally synthetized vegetable oil-based additives were tested. Three different types of technical grade vegetable oil (sunflower oil (Mw = 880 g/mol, Bunge PLC, Budapest, Hungary), rapeseed oil (Mw = 888 g/mol, Bunge PLC, Budapest, Hungary) and castor oil (Mw = 933 g/mol, Alfa Aeser, Haverhill, MA, USA) were used for additive synthesis.

### 2.2. Additive Synthesis

Vegetable oil-based additives were synthesized at the Department of MOL Hydrocarbon and Coal Processing, University of Pannonia. The synthesis of vegetable oil-based additives was carried out in a round-bottom flask equipped with a stirrer at temperature range of 130–150 °C in the presence of a hydrocarbon solvent. Stirring speed was set to be 120 rpm. The experiment was performed with three different types of vegetable oils: sunflower oil, rapeseed oil and castor oil. The molar ratio of vegetable oil to maleic anhydride was 1:1. Because of the radical initiated reactions, di-tert-butyl peroxide (supply from Merck KGaA, Darmstadt, Germany) was used. The volatiles and solvent were evaporated under vacuum at the end of reaction.

### 2.3. Sample Preparation

**Figure 1:**
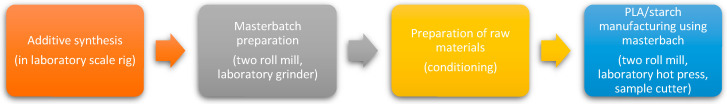
The main steps of the sample preparation.

The additives prepared by the before mentioned method were tested in PLA/starch composites in the form of a masterbatch. Masterbatches containing the synthetized compatibilizer additives were produced by a two-roll mill (LabTech Engineering Ltd., LRM-100, Praksa, Muang, Samutprakarn 10280, Thailand) at temperature range of 150–165 °C using a friction ratio of 32.8:19.3. The matrix material of the masterbatches was PLA. Figure 1 shows the main steps of the sample preparation. The starch content of blends was between 10%–50%. Before processing, all of the polymers were conditioned at 80 °C for four hours to prevent hydrolytic degradation. After homogenization of PLA, starch and masterbatch by a two-roll mill, PLA/starch sheets with size of 170 mm × 170 mm × 2 mm were formed by a laboratory hot press (CARVER 3853-0, Carver, Inc., Savannah, GA, USA) at 170 °C for ten minutes. Then 10 mm wide and 50 mm long specimens were cut out from the sheets.

### 2.4. Measurements

The main properties of vegetable oil-based additives were determined by standardized methods, by FTIR analysis (Bruker Tensor 27 instrument, USA, spectral range: 7500 to 370 cm^−1^, with a standard KBr beam-splitter, resolution: better than 1 cm^−1^ (apodised), interferometer: RockSolid, permanent aligned, high stability, sample scan time: 16 scans, background scan time: 16 scans), and through their flow properties analyzed by rheological measurements (Anton Paar MCR301 dynamic shear rheometer, Graz, Austria).

The mechanical properties of PLA/starch blends were measured from the using of INSTRON 3345 universal tensile testing machine (USA), with 75 mm/min crosshead speed for tensile tests and 5 mm/min for flexural tests. The size of specimens were 10 mm wide and 50mm long, the clambing length was 30 mm. Three parallel measurements were carried out on unconditioned (at 20 °C) and conditioned samples (at 80 °C). A CEAST Resil Impactor machine (USA, 1J hammer) with “A” type notches in both unconditioned and conditioned cases was used to know the Charpy impact strength of the samples. Furthermore, the morphology of the samples was also followed via their SEM micrographs (SEM Apreo S LoVac, Waltham, MA, USA, HV: 5–10 kV, mag: 80–20,000×).

## 3. Results

### 3.1. Additive Characterization

The main properties of the synthesized additives are summarized in Table 1. Additives had Mn in the range of 6300–8280 g/mol, while the Mw changed between 8360 and 11,910 g/mol. Additive containing castor oil had the lowest polydispersity, while that of rapeseed oil-based additive was the highest. This result refers that additive containing rapeseed oil had the most components with a different structure.

The MA-content of the additives prepared based on the three different types of vegetable oils was nearly equal (1.4–1.6 mg MA/g sample). Regarding the acid number, the additive based on sunflower oil had the most carboxyl functional groups, therefore, this additive had the highest acid number (54.1 mg KOH/g sample). On the other hand, the acid numbers of rapeseed and castor oil-based additives were almost the same (46.2 and 45.4 mg KOH/g sample). Considering the degree of unsaturation, the additive most prone to saturation was the sunflower oil-based additive, and the additional properties of rapeseed and castor oil-based additives were almost the same as the acid number.

**Figure 2:**
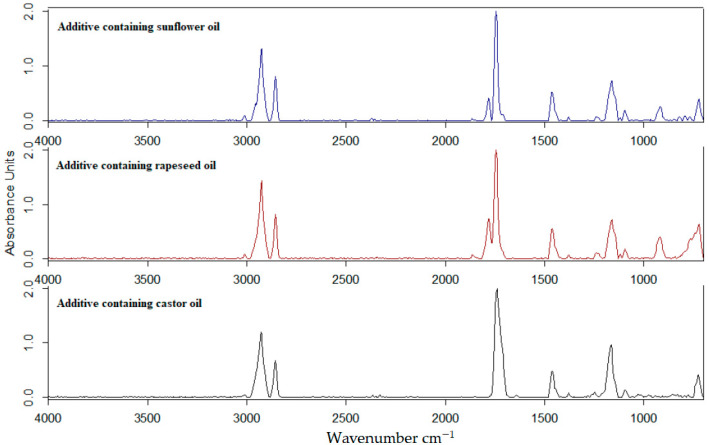
FT-IR spectra of vegetable oil-based additives in the wavenumber range 4000–600 cm^−1^.

**Figure 3:**
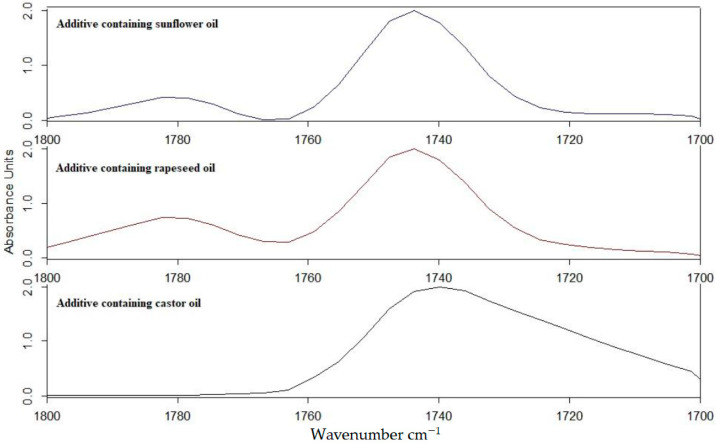
FT-IR spectra of vegetable oil-based additives in the wavenumber range 1800–1700 cm^−1^.

The FTIR spectra of the vegetable oil-based additives were determined using germanium ATR crystal. The spectra can be seen in Figure 2 and Figure 3.

In the wavenumber range of 3100–2800 cm^−1^, asymmetric and symmetric stretching vibrations of methyl and methylene groups are observed [15]. The difference between the synthesized additives is manifested in the wavenumber range of 1900–1600 cm^−1^. While in the wavenumber range of 1650–1630 cm^−1^ and 1750–1730 cm^−1^, peaks (Figure 3) appeared for all three additives as well. In the range of 1790–1770 cm^−1^, peaks appeared only in the case of rapeseed and sunflower oil-based additives. In case of the rapeseed oil, the ratio of the two peaks in the range of 1790–1740 cm^−1^ was 2.68, while in case of sunflower oil-based additive it was 3.71.

**Figure 4:**
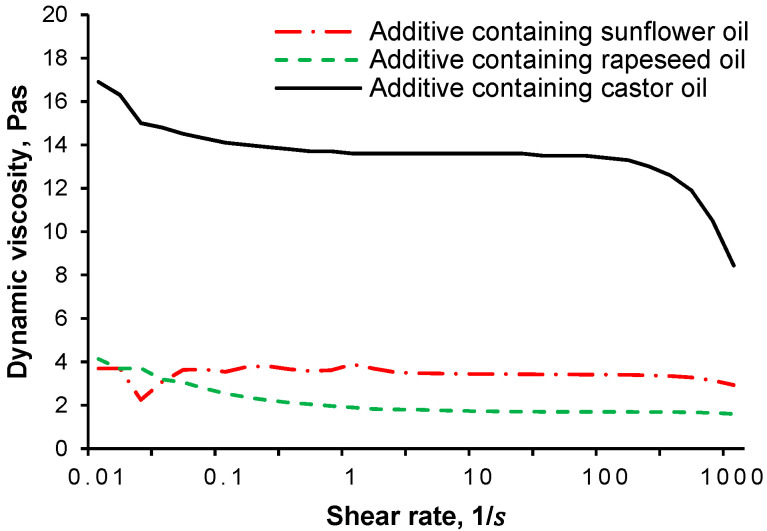
Rheological behaviour of synthesized additives in the range of 1–1000 $\frac{1}{s}$ (25 °C).

The dynamic viscosity of synthesized additives was measured at 25 °C (Anton Paar MCR301 instrument) in the shear rate range of 1–1000 $\frac{1}{s}$ as shown in Figure 4.

Based on the dynamic viscosity values, it can be stated that the additives containing castor oil had the highest dynamic viscosity, followed by the additive containing sunflower oil, and rapeseed oil. On one hand, it was caused by the effect of the length of hydrocarbon side chain of each vegetable oil. On the other hand, differences in viscosities were caused by the fact that the main components in vegetable oils are different with different structures. The main component of castor oil is ricinoleic acid which has a hydroxyl group in addition to the double bond [16]. The hydroxyl group allows maleic anhydride to be incorporated into the molecule, it allows the chemical modification of castor oil through esterification of this functional group to maleated half esters [17]. Most sunflower oils are basically linoleic acid, which have more double bonds than ricinoleic acid or oleic acid [18]. With 1 mole of unconjugated linoleic acid, maleic anhydride can form an “ene” adduct resulting in the formation of a conjugated diene, which can be further functionalized by Diels-Alder synthesis [17]. The main component of rapeseed oil is oleic acid, which has no hydroxyl group and contains less C-C double bonds than linoleic acid [19]. Oleic acid is reacted with maleic anhydride according to the “ene” reaction mechanism [17,20]. Since both rapeseed oil and sunflower oil can be found in both major components, the Diels-Alder and “ene” reaction mechanism may have occurred in both oils. This is probably the reason for the similar dynamic viscosity.

### 3.2. Masterbatch Characterization

Masterbatches were prepared by the mixing of the synthesized additives into PLA matrix. The main properties of the masterbatches are summarized in Table 2. In order to know the effect of vegetable oil-based additives to the starch-PLA composites, a masterbatch with PLA-g-MA was also prepared. The additive content of the masterbatches was 10%. Regarding the MFI values, it is clear, that PLA-g-MA had the lowest, while the sunflower oil-based masterbatch (SFO) had the highest values. However, there was no significant difference among the MFI values of the experimentally synthetized vegetable oil-based additives. Given the PLA granulates, 8.819 g/10 min MFI value was measured so it can be stated all of the vegetable oil components had softening effect, for PLA-g-MA it was not observed.

**Figure 5:**
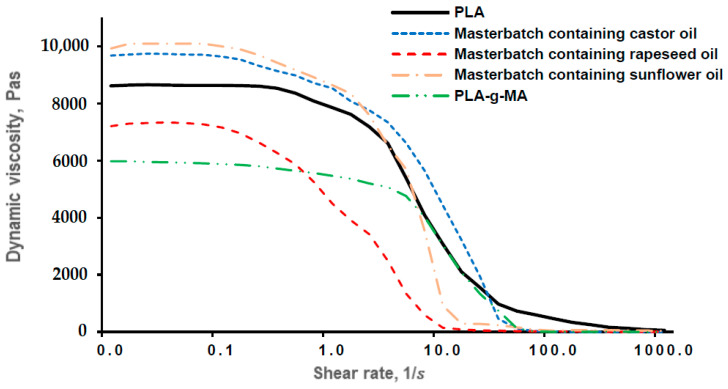
Rheological behaviour of PLA granulates and masterbatches in the range of 1–1000 $\frac{1}{s}$ (170 °C).

Figure 5 summarizes the rheological properties of the masterbatches. As it is well shown, the viscosity of masterbatches with PLA matrix did not change significantly up to the shear rate of 0.2$\frac{1}{s}$. Reaching this shear rate, the dynamic viscosity started to decrease significantly. This change leads to the conclusion that molecular formation cannot occur. Regarding the results, the masterbatch containing rapeseed oil had the largest softening effect in the measurement range.

### 3.3. Mechanical Properties of PLA/Starch Blends

The purpose of the mechanical tests on PLA/starch blends was to determine the extent to which the starch content of the blends could be increased without deteriorating their mechanical performance.

#### 3.3.1. Results

**Figure 6:**
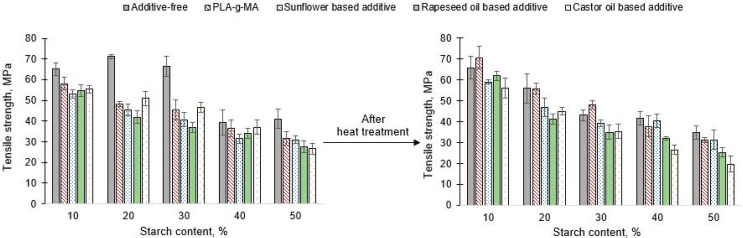
Tensile strength of unconditioned and conditioned PLA/starch blends.

Results of tensile strength (Figure 6) well shows that the tensile strength decreased with increasing starch content in both unconditioned and conditioned samples for both additive-free and additive-containing blends. In the majority of both unconditioned and conditioned cases, the tensile strength decreased as function of starch content. Comparing the conditioned specimens containing PLA-g-MA (10%–40% starch content), the tensile strength also increased compared to the unconditioned samples. In the case of unconditioned samples, the tensile strength of blends containing vegetable oil-based additives was lower than that of additive free PLA/starch samples or PLA/starch/PLA-g-MA samples. This result was presumably due to the softening effect of vegetable oil-based additives. For all tested compositions, it was observed that the PLA/starch blends without masterbatches had the highest tensile strength (from 39 to 71 MPa). It is important to mention, that at additive free PLA/starch blends had lower tensile strength after the heat treatment in any cases, while the tensile strength of PLA/starch/PLA-g-MA samples could be increased by the heat treatment. Without any heat treatment, the additive free samples had higher tensile strength than the PLA/starch blend with additive. After conditioning, specimens prepared with PLA-g-MA had higher tensile strength (about 70 and 50 MPa of blends containing 10% and 30% starch (improvement was 5 MPa compared to additive free specimens)).

**Figure 7:**
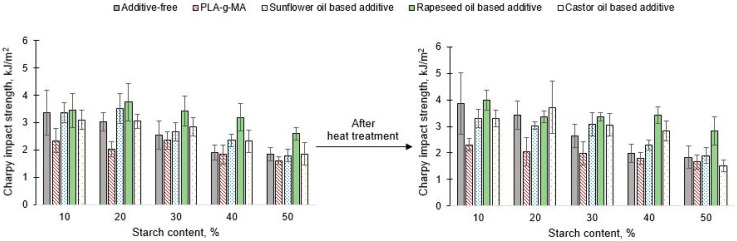
Charpy impact strength of unconditioned and conditioned PLA/starch blends.

Similar to tensile strength, it can be stated that the value of impact strength decreased with increasing starch content at both conditioning cases (Figure 7). The effect of heat treatment was more significant for samples with lower starch content (10% and 20%). To investigate the effect of masterbatches that did not contain vegetable oil-based additives, it can be concluded that the Charpy impact strength did not increase in either case. In general, the same trend was found for additive free samples, therefore, the impact strength decreased with increasing starch content. The PLA-g-MA was mostly able to compensate the negative effect of heat treatment, and no measurable difference between the two cases can be found. The positive effect of conditioning can also be observed in the case of specimens containing castor oil-based additives—in contrast to those found in the case of tensile strength—with the exception of the sample containing 50% starch, because the value of impact strength can be increased.

Regarding the unconditioned samples, specimens containing 20%, 30%, and 40% starch, higher Charpy impact strength was found with additives than without that. Same phenomena can be stated in case of conditioned samples, thus, the conditioning did not affect the role of the tested additive. In the case of unconditioned samples, the tendency was the same: the value of impact strength decreased with increasing starch content. However, in case of conditioned samples, the starch content of 20% was an exception. Regarding the samples containing rapeseed oil-based additives, it can be stated that with the exception of the samples with 20% and 30% starch, the impact strength increased as a result of heat treatment. In case of both unconditioned and conditioned samples, in general, higher impact strength can be found by the using of vegetable oil-based additives (exception PLA with 10% starch). Regarding the effect of the sunflower oil-based additives, the impact strength values were improved for samples with 30%, 40% and 50% starch content after conditioning at 80 °C and without conditioning compared to the additive free blends. Thus, to investigate the effect of any additive in based on vegetable oil, it can be concluded that for certain compositions, an improvement can be obtained for unconditioned and 80 °C conditioned specimens. An exception to this phenomenon was the additive based on rapeseed oil, since in any composition. An improvement in impact strength can be obtained by its use in both unconditioned and conditioned cases. This is because the rapeseed oil has the longest hydrocarbon chains and consequently it has higher molecular weight.

**Figure 8:**
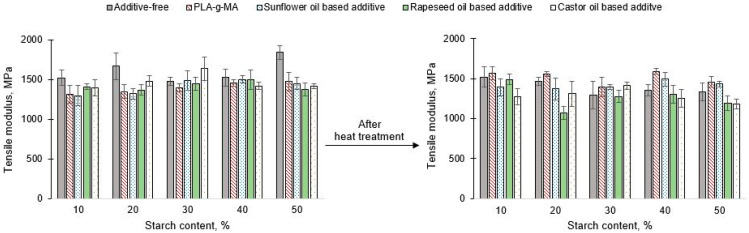
Tensile modulus of unconditioned and conditioned PLA/starch blends.

Regarding the tensile modulus (Figure 8), no trend can be established in proportion to the increase in starch content. However, the highest tensile modulus was found in case of additive free PLA/starch composites without heat treatment (in exception of 70% PLA/30% starch composites). Comparing the results, it was found, that he 50/50 PLA/starch sample (without additives) had the highest tensile modulus among the unconditioned samples. As a result of conditioning, tensile modulus can be decreased, and the highest value (about 1500 MPa) was measured on the 90/10 PLA/starch specimen. Excluding tensile modulus, the effect of heat treatment on the mechanical properties could not be compensated in case of samples containing castor oil-based masterbatch. The tensile modulus of the 70/30 PLA/starch blend could be improved in both unconditioned and conditioned cases. For the samples containing masterbatch synthetized by the using of rapeseed oil-based additives, the tensile modulus could not be improved for any of the compositions compared to the additive free samples. The depreciation, due to 80 °C conditioning did not improve either. Although the masterbatch containing sunflower oil-based additive could not compensate for the negative effect of 80 °C conditioning in all cases. It was successful at 10% and 20% starch content, and a higher tensile modulus was measured compared to the unconditioned samples. However, a lower value was found compared that of to the additive free samples. For the 60/40 and 50/50 compositions, a higher value was measured compared to the additive-free blend after the heat treatment. Similar to the 30/70 PLA/Starch samples containing castor oil-based masterbatch, the tensile modulus of this composition can be improved at both temperatures with the sunflower oil-based masterbatch. By adding the PLA-g-MA, it can be found that for almost all compositions (10%–40% starch content) the values of the tensile modulus were higher after conditioning at 80 °C, which was not observed for either the additive free blends or the samples containing the different types of vegetable oil-based additives. For the unconditioned samples, no positive effect was concluded compared to the additive free blends, however, for the samples conditioned at 80 °C, a blend of some composition showed improvement.

**Figure 9:**
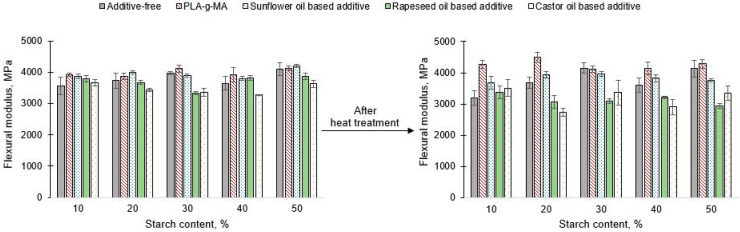
Flexural modulus of unconditioned and conditioned PLA/starch blends.

Regarding the flexural modulus (Figure 9), the unfavourable effect of conditioning also prevails, because in case of all tested compositions the values of flexural modulus after conditioning were lower.

The effect of conditioning was eliminated with only PLA-g-MA at all ratios. In addition, unconditioned samples showed a significant improvement over the flexural modulus of the additive-free PLA/starch blends (for instance 260 MPa improvement was measured in case of 90/10 PLA/starch composition). Sunflower oil-based additives also caused a significant improvement in all blends of the examined composition and the disadvantage of the heat treatment can been reduced. Overall, for both conditioned and unconditioned samples, the flexural modulus could be improved with each masterbatch in case of 10% starch content.

**Figure 10:**
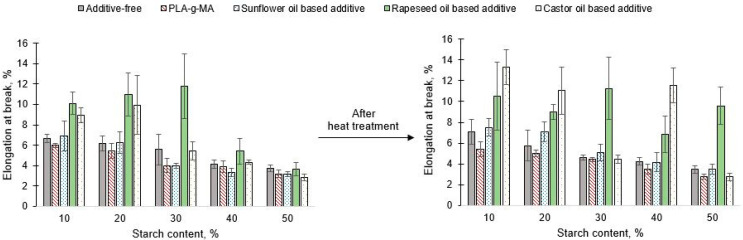
Elongation at break of unconditioned and conditioned PLA/starch blends.

Based on data in Figure 10, it was observed that increasing starch content causing a decrease in the elongation at break considering both the unconditioned and conditioned specimens. This tendency was more or less observed in the sample series containing the masterbatch. The effect of conditioning did not cause a large change in elongation at break. The largest change was a decrease from 5.6% elongation at break to 4.6% in case of additive free 30/70 PLA/starch blend. A decrease in the elongation at break was observed at both temperatures using PLA-g-MA. By using a rapeseed oil-based additive, the elongation at break could be increased regardless of composition and conditioning. Irrespective of conditioning, the castor oil and sunflower oil-based additives also resulted an increase in case of blends containing 10% and 20% starch as well.

#### 3.3.2. Summary of Mechanical Properties

One of the purposes of mechanical tests was to track changes in the mechanical properties of specimens depending on their moisture content. For this reason, the specimens were conditioned at room temperature and at the drying temperature of the raw materials (80 °C) in order to minimize the moisture content of the specimens. Ke et al. [21,22] found that the thermal and crystallization properties of PLA or the compatibility between PLA and starch were not modified significantly by moisture content. However, with increasing moisture content, the morphology of the mixtures became more uniform due to higher degree of gelatinization of the starch. Furthermore, it was found that as the starch content increased, both tensile strength and elongation at break decreased [22]. Given the mechanical properties, the type of starch has an influencing effect because different types of starch have different morphology and microstructure. The mechanical properties are significantly influenced by particle size and agglomeration of the particles, as well as a surface modification method influencing the agglomeration. [23] For example, in the experiment of Khalid et al. [23], PLA/starch composites containing large-sized starch granules showed higher strength.

The main conclusions of mechanical properties are summarized in Table 3, Table 4, Table 5 and Table 6 indicating the measured improvements and depreciations compared to the additive free PLA/starch blends.

Regarding the results of the unconditioned blends, it can be concluded that the tensile strength was the only characteristic that could not be significantly increased by adding any of the vegetable oil-based additives. Only the PLA-g-MA could slightly increase the tensile strength of the conditioned samples (90/10 and 70/30 composition). However, the PLA-g-MA cannot increase the Charpy impact strength. In contrast, all of the additives containing vegetable oil can improve the Charpy impact strength, among other things, in case of blends containing 30% and 40% starch regardless of conditioning. Regarding the tensile modulus, the blends containing PLA-g-MA showed improvements for conditioned samples (each composition), while the additives made from sunflower oil and castor oil demonstrates improvement in case of 70/30 PLA/starch composition regardless of conditioning. In terms of the improvement of flexural modulus, the PLA-g-MA clearly proved to be the most effective regardless of conditioning. However, the sunflower oil-based additive can approach the effect of PLA-g-MA for all compositions. In terms of elongation at break, blends with rapeseed oil-based additive was the most effective for all cases. In the case of blends with 10% starch content, in addition to improvement of flexural modulus, the elongation at break could also be increased by using all types of the vegetable oil-based additives, and even by using the rapeseed oil-based additive, the impact strength can be also increased.

Regarding the blends containing 20% starch, the elongation at break was higher in the case of unconditioned and conditioned specimens. In addition to elongation at break, the flexural modulus was also increased by the sunflower-based additive. In case of 30% starch content, it was found that both the Charpy impact strength and the tensile modulus can be improved by using the castor oil or sunflower oil-based additives as well, while by using of rapeseed oil-based additive, the impact strength and elongation at break were increased in the temperature range of 25–80 °C. For 60/40 PLA/starch blend, the impact strength could be increased with all three vegetable oil-based additives, and the flexural modulus was improved by using of sunflower oil-based additives. Furthermore, the value of the elongation at break can be also increased due to the additive based on castor oil and rapeseed oil. Finally, regarding the samples containing 50% starch, no improvement could be obtained using castor oil-based additive in any of the properties, but the value of impact strength and elongation at break were better with the use of rapeseed oil-based additive, while the flexural modulus was increased as well using sunflower oil-based additive.

### 3.4. Morphological Examination of the Structure

**Figure 11:**
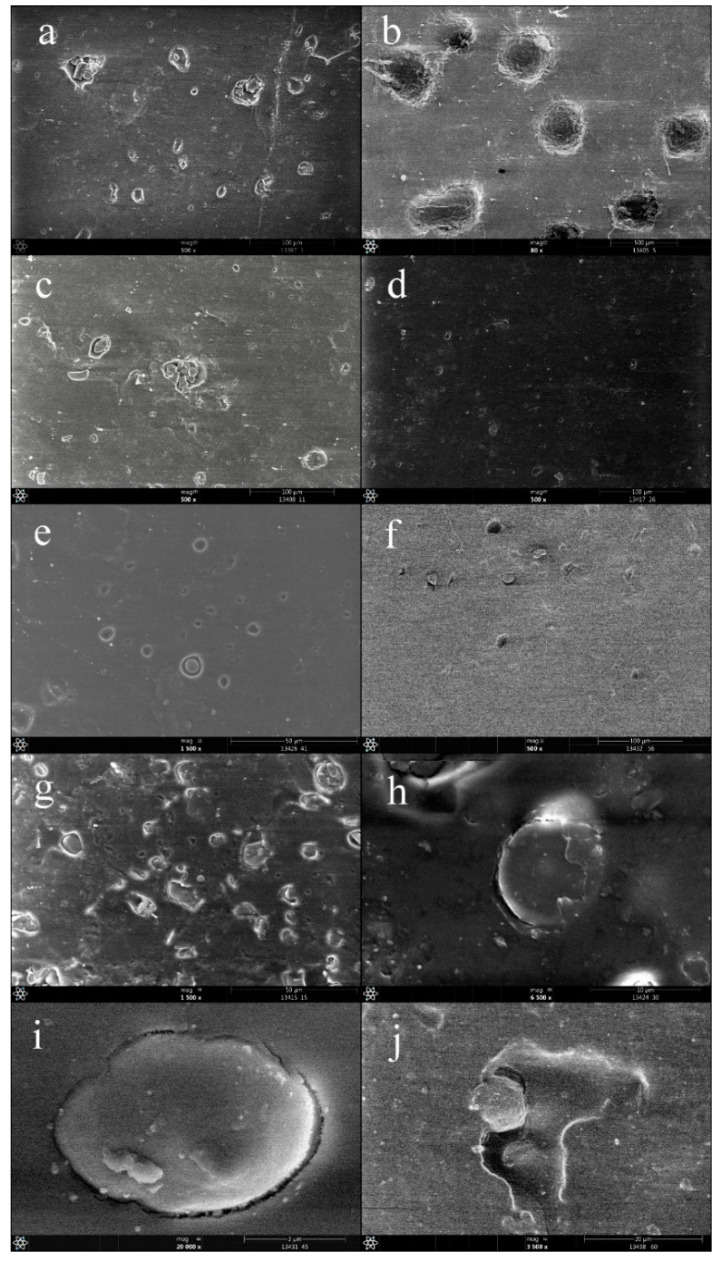
SEM images of compatibilized and uncompatibilised PLA/starch blends containing 10 and 50% starch ((**a**) 90/10 PLA/starch without compatibilization, (**b**) 50/50 PLA/starch without compatibilization, (**c**) 90/10 PLA/starch with castor oil-based masterbatch, (**d**) 90/10 PLA/starch with rapeseed oil-based masterbatch, (**e**) 90/10 PLA/starch with sunflower oil-based masterbatch, (**f**) 90/10 PLA/starch with PLA-g-MA, (**g**) 50/50 PLA/starch with castor oil-based masterbatch, (**h**) 50/50 PLA/starch with rapeseed oil-based masterbatch, (**i**) 50/50 PLA/starch with sunflower oil-based masterbatch, (**j**) 50/50 PLA/starch with PLA-g-MA).

Morphological structures of samples containing 10% and 50% starch content were investigated by scanning electron microscopy (SEM) (Figure 11).

The aim of this investigation was to determine whether the morphological structure was modified by adding masterbatch, to see if whether the compatibility was effective. Regarding the morphology of PLA/starch blends with composition of 90/10 and 50/50 without any additives (Figure 11a–c), it can be stated that the mutual miscibility of the two phases is only partial regardless of whether they contain 10% or 50% starch. Starch can be observed as a dispersed phase and PLA as a matrix. The images clearly show phase separation, in addition the starch particles were unevenly dispersed in the PLA matrix. The 50/50 PLA/starch blend has a more uneven structure than 90/10 blend. Regarding the morphology of the samples with 90/10 composition (Figure 11a,c–f), it was found that the most uniform surface is available in case of blend containing rapeseed oil-based additive. Given the blend containing castor oil-based additive, the separation of dispersed phases (starch) from the matrix was observed. The structure of the sample containing sunflower oil-based additive appears to be smoother than that of the sample containing castor oil, however, the separation of the dispersed starch phase is also evident in the SEM images. Although the structure is smoother for samples containing the PLA-g-MA masterbatch, the dispersed phase is also more or less separated from the PLA. Overall, compared to the additive-free samples, with the exception of the blend containing castor oil, a more uniform appearance of the morphology was observed in all cases, the distribution and the incorporation of the dispersed phase into the matrix could be improved. In case of sample containing 50% starch, the starch phases are completely separated from the matrix material resulting in a severe inhomogeneous morphology. Two important changes can be observed in the structure of the samples containing the additives: one is that the dispersed phases have smaller sizes and less agglomeration and are more evenly distributed. The other is that the matrix material can involve better than the dispersed phases. This positive effect is most pronounced for samples containing castor oil and least for blends containing PLA-g-MA masterbatch.

## 4. Conclusions

The aim of the experiment was to improve the compatibility between PLA and starch. To improve miscibility, PLA-g-MA and vegetable oil-based additives containing maleic anhydride were used in the form of masterbatches. The heat treatment at 80 °C has caused significant differences in mechanical properties, the effect of which could be eliminated in some samples by adding the synthesized additive. The effect of vegetable oil-based additives was most pronounced in Charpy impact strength. Using rapeseed oil-based additive, improvements were observed compared for several specimens to the additive-free specimens, for instance, in case of 50/50 PLA/starch blend, there was a nearly 40% improvement in the impact strength for the unconditioned sample, and a nearly 50% improvement after heat treatment compared to the additive-free blend. SEM images also confirmed the advantageous effect of the rapeseed oil-based additive. The effect of PLA-g-MA on the Charpy impact strength was not favourable. Taking into account the results of tensile tests, it was reduced in all cases, and the tensile modulus could be improved in case of 70/30 PLA/starch composition using castor oil and sunflower oil. The rate of improvement was about 10% both before and after the heat treatment. In terms of flexural modulus, PLA-g-MA proved to be more effective against vegetable oil-based additives.

## Figures and Tables

**Table 1:** Acid number, iodine-bromide number and MA-content values of synthesized additives.

Properties	Sunflower Oil	Sunflower Oil-Based Additive	Rapeseed Oil	Rapeseed Oil-Based Additive	Castor Oil	Castor Oil-Based Additive
Mw, g/mol	-	8360	-	9680	-	11,910
Mn, g/mol	-	6300	-	6570	-	8280
Polydispersity	-	1.30	-	1.36	-	1.25
Acid number, mg KOH/g sample	3.3	54.1	8.6	46.2	2.9	45.4
Ionide-bromide number, I_2_/100 g sample	106.7	84.6	101.2	75.5	93.3	75.5
MA-content, mg MA/g sample	-	1.4	-	1.5	-	1.6

**Table 2:** The main properties of masterbatches (MB).

Properties	SFO-MB	RSO-MB	CO-MB	PLA-g-MA
Active agent	Sunflower oil-based additive	Rapeseed oil-based additive	Castor oil-based additive	PLA-g-MA
Additive content, %	10	10	10	10
MFI, g/10min (5.00 kg, 190 °C)	10.7	10.3	10.1	8.6

**Table 3:** Effects on mechanical properties of castor oil-based additive (“+”: positive change relative to an additive-free blend of a suitable composition; “−”: negative change relative to an additive-free blend of a suitable composition).

Starch Content, %	Unconditioned Blends					Conditioned Blends				
	Tensile Strength	Charpy Impact Strength	Tensile Modulus	Flexural Modulus	Elongation at Break	Tensile Strength	Charpy Impact Strength	Tensile Modulus	Flexural Modulus	Elongation at Break
10	−	−	−	+	+	−	−	−	+	+
20	−	−	−	−	+	−	+	−	−	+
30	−	+	+	−	−	−	+	+	−	+
40	−	+	−	−	+	−	+	−	−	+
50	−	−	−	−	−	−	−	−	−	−

**Table 4:** Effects on mechanical properties of rapeseed oil-based additive (“+”: positive change relative to an additive-free blend of a suitable composition; “−”: negative change relative to an additive-free blend of a suitable composition).

Starch Content, %	Unconditioned Blends					Conditioned Blends				
	Tensile Strength	Charpy Impact Strength	Tensile Modulus	Flexural Modulus	Elongation at Break	Tensile Strength	Charpy Impact Strength	Tensile Modulus	Flexural Modulus	Elongation at Break
10	−	+	−	+	+	−	+	−	+	+
20	−	+	−	−	+	−	−	−	−	+
30	−	+	−	−	+	−	+	−	−	+
40	−	+	−	+	+	−	+	−	−	+
50	−	+	−	−	+	−	+	−	−	+

**Table 5:** Effects on mechanical properties of sunflower oil-based additive (“+”: positive change relative to an additive-free blend of a suitable composition; “−”: negative change relative to an additive-free blend of a suitable composition).

Starch Content, %	Unconditioned Blends					Conditioned Blends				
	Tensile Strength	Charpy Impact Strength	Tensile Modulus	Flexural Modulus	Elongation at Break	Tensile Strength	Charpy Impact Strength	Tensile Modulus	Flexural Modulus	Elongation at Break
10	−	−	−	+	+	+	−	−	+	+
20	−	+	−	+	+	−	−	−	+	+
30	−	+	+	−	−	+	+	+	+	+
40	−	+	−	+	−	−	+	+	+	−
50	−	−	−	+	−	−	+	+	+	−

**Table 6:** Effects on mechanical properties of PLA-g-MA (“+”: positive change relative to an additive-free blend of a suitable composition; “−”: negative change relative to an additive-free blend of a suitable composition).

Starch Content, %	Unconditioned Blends					Conditioned Blends				
	Tensile Strength	Charpy Impact Strength	Tensile Modulus	Flexural Modulus	Elongation at Break	Tensile Strength	Charpy Impact Strength	Tensile Modulus	Flexural Modulus	Elongation at Break
10	−	−	−	+	−	+	−	+	+	−
20	−	−	−	+	−	−	−	+	+	−
30	−	−	−	+	−	+	−	+	+	−
40	−	−	−	+	−	−	−	+	+	−
50	−	−	−	+	−	−	−	+	+	−

## Data Availability

Not applicable.
